# The Bonwill Dental Engine and Mallet

**Published:** 1887-06

**Authors:** 


### THE BONWILL DENTAL ENGINE AND MALLET.

Dr. W. G. A. Bonwill has sold the patterns and the right to manufacture his dental engine and mechanical mallet to The S. S. White Dental Manufacturing Co., and they will henceforth have the sole control of them. In some respects his dental engine is superior to any that is manufactured, while his mechanical mallet is capable of producing as beautiful and perfect results as anything of the kind that is known to dentists. Dr. Bonwill is an ingenious man, and that his inventions have been meritorious is proved by their sale, which, during the past fifteen years, has, we are informed, amounted to over $60,000, and he now receives more than half as much more in their final disposal.

They are few avocations which afford greater scope to ingenious men than does dentisty. The results at which the practitioner aims may be accomplished by so many varying methods, and mechanical science is so large a factor in most dental operations, that inventive genius is constantly stimulated to its highest accomplishments. So many mechanical implements are also employed by the dentist, and the efficiency of his work is so largely dependent upon the perfection of his instruments, that ample room is afforded for constant improvement in this field.

The propriety of securing a monopoly of manufacture by patenting the various devices and improvements made, has long been a vexed question in dentisty, but we believe that few object to remunerating the frequently protracted studies and long course of experimentation necessary to perfect an invention, by paying a reasonable royalty upon any really meritorious and original device. It is the taking out of patents upon trivial modifications, too often the securing of them upon devices and methods as old as dentisty itself, against which practitioners protest. One feels outraged when a claim is made for royalty upon and damages for use of some device which he has employed for many years, but for the essential points of which some dental pirate has secured a patent in secrecy and by stealth. Such claims are scarcely worth contesting, for it is usually cheaper to pay the amount than to fee lawyers. The inventions of Dr. Bonwill do not belong to this class, and all will rejoice that he has received a fair reward for the time and labor spent in perfecting them.

